# Mpox vaccination willingness, determinants, and communication needs in gay, bisexual, and other men who have sex with men, in the context of limited vaccine availability in the Netherlands (Dutch Mpox-survey)

**DOI:** 10.3389/fpubh.2022.1058807

**Published:** 2023-01-05

**Authors:** Nicole H. T. M. Dukers-Muijrers, Ymke Evers, Veja Widdershoven, Udi Davidovich, Philippe C. G. Adam, Eline L. M. Op de Coul, Paul Zantkuijl, Amy Matser, Maria Prins, Henry J. C. de Vries, Casper den Heijer, Christian J. P. A. Hoebe, Anne-Marie Niekamp, Francine Schneider, Juliana Reyes-Urueña, Roberto Croci, Angelo D'Ambrosio, Marc van der Valk, Dirk Posthouwer, Robin Ackens, Henriette ter Waarbeek, Teymur Noori, Elske Hoornenborg

**Affiliations:** ^1^Department of Sexual Health, Infectious Diseases, and Environmental Health, South Limburg Public Health Service, Heerlen, Netherlands; ^2^Department of Health Promotion, Care and Public Health Research Institute (CAPHRI), Maastricht University, Maastricht, Netherlands; ^3^Department of Social Medicine, Care and Public Health Research Institute (CAPHRI), Maastricht University, Maastricht, Netherlands; ^4^Department of Infectious Diseases, Public Health Service of Amsterdam (GGD Amsterdam), Amsterdam, Netherlands; ^5^Department of Social Psychology, University of Amsterdam, Amsterdam, Netherlands; ^6^Institute for Prevention and Social Research in Utrecht, Utrecht, Netherlands; ^7^Centre for Social Research in Health, University of New South Wales (UNSW) Sydney, Kensington, NSW, Australia; ^8^Epidemiology and Surveillance Unit, Centre for Infectious Disease Control, National Institute for Public Health and the Environment (RIVM), Bilthoven, Netherlands; ^9^Soa Aids Nederland, Amsterdam, Netherlands; ^10^Amsterdam University Medical Center (UMC) Location University of Amsterdam, Infectious Diseases, Amsterdam, Netherlands; ^11^Amsterdam Institute for Infection and Immunity (AII), Amsterdam UMC Location University of Amsterdam, Amsterdam, Netherlands; ^12^Department of Dermatology, Location Academic Medical Centre, Amsterdam Institute for Infection and Immunity (AII), Amsterdam UMC, University of Amsterdam, Amsterdam, Netherlands; ^13^Department of Medical Microbiology, Care and Public Health Research Institute (CAPHRI), Maastricht University Medical Center (MUMC+), Maastricht, Netherlands; ^14^European Centre for Disease Prevention and Control (ECDC), Stockholm, Sweden; ^15^Stichting HIV Monitoring, Amsterdam, Netherlands; ^16^Department of Internal Medicine, Care and Public Health Research Institute (CAPHRI), Maastricht University Medical Center (MUMC+), Maastricht, Netherlands; ^17^Department of Integrated Care, Maastricht University Medical Centre (MUMC+), Maastricht, Netherlands

**Keywords:** vaccination, communication, GBMSM, mpox, public health, prevention, low urban, social network

## Abstract

**Introduction:**

In the 2022 multicountry mpox (formerly named monkeypox) outbreak, several countries offered primary preventive vaccination (PPV) to people at higher risk for infection. We study vaccine acceptance and its determinants, to target and tailor public health (communication-) strategies in the context of limited vaccine supply in the Netherlands.

**Methods:**

Online survey in a convenience sample of gay, bisexual and other men who have sex with men, including transgender persons (22/07-05/09/2022, the Netherlands). We assessed determinants for being (un)willing to accept vaccination. We used multivariable multinominal regression and logistic regression analyses, calculating adjusted odds ratios (aOR) and 95 percent confidence-intervals. An open question asked for campaigning and procedural recommendations.

**Results:**

Of respondents, 81.5% (*n* = 1,512/1,856) were willing to accept vaccination; this was 85.2% (799/938) in vaccination-eligible people and 77.7% (713/918) in those non-eligible. Determinants for non-acceptance included: urbanization (rural: aOR:2.2;1.2–3.7; low-urban: aOR:2.4;1.4–3.9; vs. high-urban), not knowing mpox-vaccinated persons (aOR:2.4;1.6–3.4), and lack of connection to gay/queer-community (aOR:2.0;1.5–2.7). Beliefs associated with acceptance were: perception of higher risk/severity of mpox, higher protection motivation, positive outcome expectations post vaccination, and perceived positive social norms regarding vaccination. Respondents recommended better accessible communication, delivered regularly and stigma-free, with facts on mpox, vaccination and procedures, and other preventive options. Also, they recommended, “vaccine provision also at non-clinic settings, discrete/anonymous options, self-registration” to be vaccinated and other inclusive vaccine-offers (e.g., also accessible to people not in existing patient-registries).

**Conclusion:**

In the public health response to the mpox outbreak, key is a broad and equitable access to information, and to low-threshold vaccination options for those at highest risk. Communication should be uniform and transparent and tailored to beliefs, and include other preventive options. Mpox vaccine willingness was high. Public health efforts may be strengthened in less urbanized areas and reach out to those who lack relevant (community) social network influences.

## 1. Introduction

Mpox (formerly named monkeypox) outbreaks have been reported in non-endemic countries since May 2022 (1–3). Since the start until 6 December 2022, 82,088 confirmed cases of mpox, and 64 deaths, were reported worldwide, and 20,934 mpox cases from 29 EU/EEA countries, including 1,251 in the Netherlands (3, 4). Compared to the peak of reported cases (2,164 cases during week 29; 18–24 July 2022), there has been a decrease in the number of newly reported cases (by 98.8% during week 44) (3). Mpox cases during the outbreak were associated with transmission by sexual and intimate contact, and gay, bisexual and other men who have sex with men and transgender persons (GBMSM/TGP) who had multiple sexual partners had been disproportionally affected (1, 2). On July 23, 2022, the WHO Director-General declared the escalating global mpox outbreak a Public Health Emergency of International Concern (5). To address these outbreaks with the required urgency, countries applied preventive measures as active case finding, contact tracing, self-isolation, and quarantine. As mpox is caused by a virus similar to smallpox, smallpox vaccines are expected to prevent or reduce the severity of the mpox infection and onward transmission (6–8). Smallpox vaccine development has a long history, and during the outbreak, various countries had offered these vaccines as post-exposure vaccination (PEPV) to contacts of a mpox case and as primary preventive pre-exposure vaccination (PPV) (9). Scarce vaccine supplies challenge an equitable global and national public health response (10). Countries that do have available vaccines, including the Netherlands, have limited vaccine supply and did restrict access to PPV based on high risk of mpox exposure (11). To achieve a high vaccination coverage in people who have a high risk for exposure is important to control the spread of mpox. People's willingness to accept PPV is a vital step. An online survey in the European region conducted during the peak of the outbreak, in 32,902 users of gay dating apps, demonstrated high willingness to accept PPV in Northern [84.8–90.4%] and Western Europe [83.1–87.7%] and somewhat lower in South-Eastern [60.9–70.2%] and Eastern Europe [59.9–71.1%] (12).

For the design of successful public health strategies, it is key to know the relevant determinants for vaccine acceptance (13–15). Some of these determinants can inform the targeting of public health efforts to subgroups that have lower PPV acceptance. Other theory-based determinants can reflect the underlying beliefs for PPV acceptance and thereby can inform the tailoring of communication-messages.

We conducted an online survey in mpox unvaccinated GBMSM/TGP, around the start of the mpox PPV-program in the Netherlands (16). The survey evaluates willingness to accept PPV and its determinants, and it also asked persons for their recommendations in campaigning and PPV-program procedures. These insights will contribute to preparing for and the shaping of an equitable and inclusive public health response to an infectious diseases outbreak.

## 2. Methods

### 2.1. Setting in the Netherlands

PEPV was already available at an earlier stage and the PPV program started at 25 July 2022 (11). The most affected large cities started, and in August, PPV activities were gradually rolled out throughout the country. A total number of vaccine doses were available to invite 32,000 people by personal email or letter, based on patient-registries of public health Center for Sexual Health (CSH), HIV outpatient clinics, or general practitioners (GP). PPV eligible were GBMSM/TGP participating in (or on a waiting list for) the national pre-exposure prophylaxis program for HIV (HIV-PrEP), were living with HIV and deemed at mpox risk by the HIV-nurse, or had according to a CSH registry in the past 6 months a sexually transmitted infection (STI) diagnosis (syphilis, gonorrhea, or chlamydia), were notified for STI/HIV, or had more than three sex partners (11).

### 2.2. Study design and recruitment

A prospective online survey of which baseline data are reported (cross-sectional design). Respondents were recruited by convenience sampling *via* social media channels and “offline” at CSH, HIV outpatient clinics, and sex-on-premises venues. Details on recruitment channels are available in Supplementary material 1. Recruitment was from 22 July to 5 September 2022, around the early roll-out of PPV in the Netherlands. During the survey-period, vaccination was by subcutaneous route of administration.

### 2.3. Participation, ethical approval, and study population

**Figure 1:**
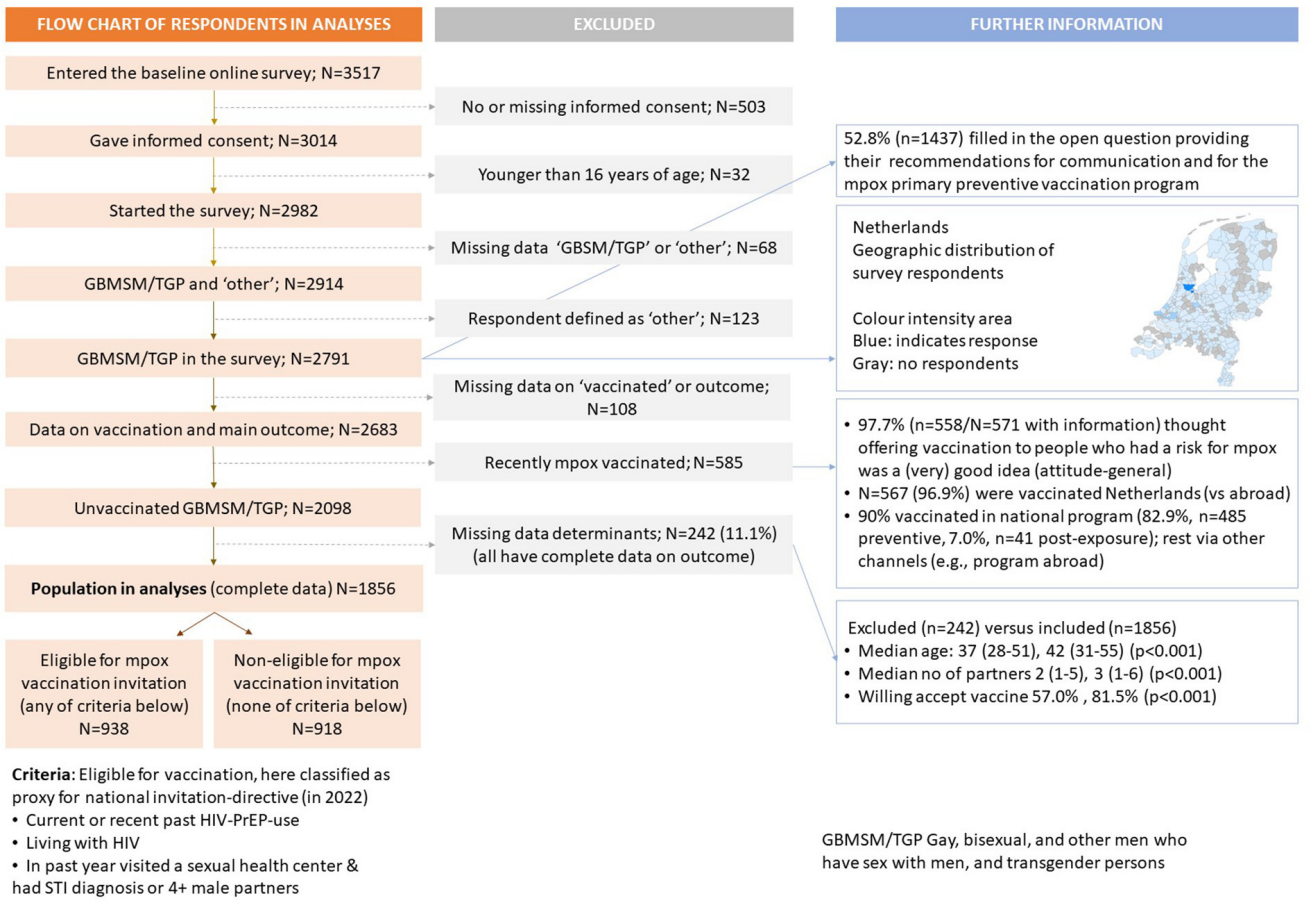
Flow chart of the number of respondents in the Dutch online mpox-survey.

People who were 16 years or older were eligible for participation. Participation started after providing informed consent to the study. Consent was also asked to respondents whether they agreed to be approached for later follow-up. The Medical Ethical Committee of Maastricht University waived ethical approval because the data were coded and were analyzed anonymously (METC 2022-3324). People who (ever) had sex with a man were included in analyses when they also reported (i) male sex or intersex and male, non-binary, genderfluid, or agender gender identity (GBMSM), or (ii) male sex and female gender identity, or female sex and male gender identity (TGP). See Figure 1 for the flowchart of included persons in the study population.

### 2.4. Online questionnaire

The questionnaire was available in Dutch and English, and its development was informed by a community consultation. Details on variables and their order (e.g., the questionnaire assessed willingness at the start) are available in Supplementary material 2.

#### 2.4.1. Main outcome

Intention is operationalized as willingness to accept PPV when offered, by the statement “If you could receive a vaccine against mpox, would you get vaccinated against mpox?” with response options 1–5 (Likert scale), “No, certainly not,” “No, probably not,” “Neutral,” “Yes, probably,” “Yes, certainly.” As a secondary outcome, willingness to accept PEPV was assessed (same Likert scale) by the statement “Suppose you had sex with someone with mpox, would you get vaccinated?”.

#### 2.4.2. Determinants for the targeting of strategies

Sociodemographic, medical, social environment factors, and behaviors were considered as important determinants to inform the targeting of strategies to determinant-subgroups with higher PPV non-acceptance.

#### 2.4.3. Determinants for tailoring strategies

Socio-cognitive determinants such as attitudes, cognitions, and perceptions (here called: beliefs) may influence willingness to accept vaccination (17–21). Beliefs are reputed modifiable by tailored communication-messages. Beliefs were presented in Supplementary material 2, and include (i) perceived risk, severity, and concern about mpox, (ii) motivation/perceived importance to protect against mpox, (iii) perceived response efficacy of vaccination, and (iv) perceived norms/social influence. Beliefs were selected based on their relevance at the time of survey-design before PPV-program start. All were theory-based derived from the Protection Motivation Theory, Health belief model, and Theory of Planned Behavior (18–20).

#### 2.4.4. Open question on campaigning and procedural recommendations

To collect non-guided insights in mpox communication and procedural preferences from GBMSM/TPG participants, an open question was included stating “What do you think is important in communication about vaccination against Mpox? You can, for example, indicate what and how, in your opinion, organizations can best communicate about this to people, or where you would like to get the vaccine.”

### 2.5. Eligible to receive invitation for vaccination in the Dutch PPV

Respondents were categorized as likely PPV “eligible” or “non-eligible,” which is a best proxy for the actual Dutch directive for PPV-eligibility (11). Categorization was based on self-reported information only (not clinic registry information) and based on similar criteria as in the directive (exact criteria were not known at the time of survey-design) (11). Respondents were categorized as “eligible” when they reported (i) HIV-PrEP use in the past 3 months or longer ago, (ii) living with HIV (regardless of antiretroviral therapy use or sexual behavior), or (iii) visited a CSH in the past year and reported a diagnosis of chlamydia, gonorrhea, or syphilis in the past year or reported more than three male sex partners in the past 3 months. All other respondents not reporting any of these criteria were categorized as PPV non-eligible.

### 2.6. Statistical analyses

#### 2.6.1. Quantitatively measured data

Analyses were performed for the entire group, and for eligible and non-eligible respondents. Firstly, descriptive statistics were provided on study population characteristics. Secondly, we provided descriptive statistics on the outcomes of this study, by its five categories and a priori regrouping into three categories, i.e., willing to accept PPV (certainly willing and likely willing), neutral, and unwilling (likely and certainly not willing). Thirdly, we evaluated non-modifiable determinants that could serve as targets of prevention strategies. Evaluated were categorical sociodemographic, medical, social environment, and behavior factors (Supplementary material 2). Using univariable multinominal logistic regression analyses, the odds for being unwilling or neutral were expressed for each of the variable-categories compared to the reference category, calculating odds ratios (ORs) and 95 percent confidence intervals (CI). For multivariable analyses, we used a bidirectional stepwise procedure to identify the most important determinants. The bidirectional stepwise procedure started by a forward approach followed by backward elimination (variables with p<0.05 could stay in the model) and repeating these steps (including initial variables) until no new variables were added. This procedure was performed for the entire study population, those eligible and those non-eligible for PPV. In sensitivity analyses, we added calendar week and channel of recruitment to the multivariable models and these recruitment factors appeared not associated (Supplementary material 3). Fourthly, we aimed to identify key beliefs (Supplementary material 2) to inform tailoring of communication-strategies. In those eligible and in those non-eligible for PPV, we used univariable logistic regression analyses, expressing the odds of being willing to accept vaccination, for each point increase on the beliefs-scores. As aim was to assess all relevant modifiable targets to inform tailoring of communications, a separate multivariable model was constructed for each belief, adjusting for identified important non-modifiable determinants. Across analyses, we considered a *P* < 0.05 as statistically significant. All analyses were performed using SPSS package vs24 (IBM Corp., Armonk, New York, USA).

#### 2.6.2. Open question on recommendations, and qualitative data

Answers were assessed (ND, YE) and categorized into main themes that arose from the answers (inductive coding). Saturation in the answers had been reached. Comments were described per theme and illustrated with examples of citations, and where possible linked to theoretical evaluated beliefs and other determinants (deductive coding).

## 3. Results

### 3.1. Respondents

Of the 2,683 GBMSM/TGP respondents, 2,098 were unvaccinated for mpox of whom 1,856 (88.5%) completed the survey and 242 not completed the survey. This latter group was younger, had fewer recent sex partners, and were more often unwilling/neutral to PPV acceptance, compared to those who completed the survey (Supplementary material 4).

### 3.2. Characteristics of the study population

Of 1,856 unvaccinated respondents included in analyses, 84% were born in the Netherlands, 23% lived in moderate urban to rural areas, 25% had a low/medium educational level, and median age was 42 (Table 1).

**Table 1:** Characteristics of the study population who are unvaccinated for mpox; GBMSM/TGP participating in Dutch online mpox-survey (22 July-5 Sept 2022).

**Characteristics, see for explanation of all categories Supplementary material 2**	**Total % (*n/N* = 1,856)**	**Eligible for vaccination & % (*n/N* = 938)**	**Non-eligible for vaccination & % (*n/N* = 918)**
**Sociodemographics**			
**Country of birth**			
The Netherlands	84.2 (1,563)	82.4 (773)	86.1 (790)
Other	15.8 (293)	17.6 (165)	13.9 (128)
**Residence country**			
The Netherlands	97.9 (1,817)	98.0 (919)	97.8 (898)
Other	2.1 (39)	2.0 (19)	2.2 (20)
**Residence level of urbanization**			
Rural areas	6.3 (117)	3.9 (37)	6.6 (61)
Hardly urbanized areas	7.4 (137)	6.0 (56)	7.8 (72)
Moderately urbanized areas	9.5 (176)	8.4 (79)	10.6 (97)
Strongly or extremely urbanized areas	20.6 (383)	19.8 (186)	21.5 (197)
Extremely urbanized areas	52.5 (975)	54.9 (515)	50.1 (460)
Unknown/abroad	3.7 (68)	3.9 (37)	3.4 (31)
**Residence SES score neighborhood**			
Low	32.7 (607)	32.8 (308)	32.6 (299)
Middle	32.0 (594)	29.9 (280)	34.2 (314)
High	30.5 (567)	32.2 (302)	28.9 (265)
Unknown	4.7 (88)	5.1 (48)	4.4 (40)
**Personal received education**			
Low	6.5 (120)	6.8 (64)	6.1 (56)
Medium	18.9 (350)	18.9 (177)	18.8 (173)
High	74.7 (1,386)	74.3 (697)	75.1 (689)
**Age** (in years) median and IQR	42 (31–55)	44 (33–55)	40 (29–55)
**Medical factors**			
**Used HIV-PrEP**			
No	73.6 (1,366)	47.8 (448)	100.0 (918)
Yes, in past 3 months or longer ago	26.4 (490)	52.2 (490)	0.0 (0)
**HIV status**			
Negative/not tested or non-disclose	87.9 (1,631)	76.0 (713)	100.0 (918)
Positive *(96% were on ART)*	12.1 (225)	24.0 (225)	0.0 (0)
**STI in past year**			
No/don't know	81.3 (1,509)	66.5 (624)	96.4 (885)
Yes	18.7 (347)	33.5 (314)	3.6 (33)
**Visited CSH in past year**			
No	54.2 (1,006)	27.3 (256)	81.7 (750)
Yes	45.8 (850)	72.7 (682)	18.3 (168)
**Vaccinated small pox**			
No/don't know	54.7 (1,015)	53.8 (505)	55.6 (510)
Yes	45.3 (841)	46.2 (433)	44.4 (408)
**Overall rated health**			
Not good/neutral	17.1 (317)	15.6 (146)	18.6 (171)
(very) good	82.9 (1,539)	84.4 (792)	81.4 (747)
**Had mpox since May 2022**			
No/don't know	97.6 (1,811)	96.2 (902)	99.0 (909)
Yes	2.4 (45)	3.8 (36)	1.0 (9)
**Social environment**			
**Know someone with mpox**			
No	81.1 (1,506)	71.9 (674)	90.6 (837)
Yes	18.9 (350)	28.1 (264)	9.4 (86)
**Know someone who had been vaccinated against mpox**			
No	62.3 (1,157)	54.9 (515)	69.9 (642)
Yes	37.7 (699)	45.1 (423)	30.1 (27.6)
**Interpersonal trust (have many people I can trust)**			
Not (completely agree)	31.7 (588)	31.9 (299)	31.5 (289)
(Completely) agree	68.3 (1,268)	68.1 (639)	68.5 (629)
**Share of MSM in friend-social network**			
None/some/neutral	51.2 (950)	43.1 (404)	59.5 (546)
(very) large part	48.8 (906)	56.9 (534)	40.5 (372)
**Connectedness to gay/queer-community**			
Not connected/neutral	43.0 (798)	38.1 (357)	48.0 (441)
Connected	57.0 (1,058)	61.9 (586)	52.0 (477)
**Behavior**			
**Close physical contact with others in work or sports**			
No	76.6 (1,422)	75.5 (708)	77.8 (714)
Yes	23.4 (434)	24.5 (230)	22.1 (204)
**Sex with men and women**			
Only with men	95.7 (1,777)	97.1 (911)	94.4 (866)
(also with) women in past 3 months	4.3 (79)	2.9 (27)	5.7 (52)
Number of male sex partners past 3 months (median and IQR)	3 (1–6)	5 (3–10)	2 (1–4)
**Anal sex with man without condom in past 3 months**			
No	34.5 (640)	21.5 (202)	47.7 (438)
Yes, with steady partner only	22.4 (415)	15.6 (146)	29.3 (269)
Yes, (also) with casual sex partners	43.2 (801)	62.9 (590)	23.0 (211)
**Practiced group sex in past 3 months with men**			
No/not disclose	73.0 (1,355)	61.2 (574)	85.1 (781)
Yes	27.0 (501)	38.8 (364)	14.9 (137)
**Chemsex in past 3 months**			
No/not disclose	75.9 (1,409)	64.4 (604)	87.7 (805)
Yes	24.1 (447)	35.6 (334)	12.3 (113)
**Received money or goods in exchange for sex past 3 months**			
No/not disclose	98.0 (1,818)	97.0 (910)	98.9 (908)
Yes	2.0 (38)	3.0 (28)	1.1 (10)
**Sexual identity**			
GBMSM	98.7 (1,831)	98.9 (928)	98.4 (903)
TPG	1.3 (25)	1.1 (10)	1.6 (15)

& based on information in the questionnaire, which is a proxi for the Dutch mpox vaccination program criteria (at time of questionnaire 22 July-5 Sept 2022).

Of 938 PPV eligible respondents, 52% used HIV-PrEP and 24% were living with HIV (96% used ART) (Table 1). 28% knew someone who had mpox, 45% knew someone who was vaccinated against mpox, 38% lacked connectedness to the gay/queer-community, and over the past 3 months 40% reported group sex, 66% reported unprotected anal intercourse (UAI) during casual sex, and 70% reported more than three sex partners.

Of 918 PPV non-eligible respondents (HIV negative/untested; none used HIV-PrEP), 82% not attended a CSH in the past year, 48% lacked connectedness to the gay/queer-community, and over the past 3 months 15% reported group sex, 23% reported UAI during casual sex, 25% reported more than three sex partners (none engaged in SHC care), and 12% used drugs during sex.

### 3.3. Willingness to accept vaccination

Of respondents, 81.5% were willing to accept vaccination; this was 85% in those PPV eligible and 78% in those non-eligible (Table 2). Of respondents, 12% were unwilling to accept vaccination; this was 10% in those eligible and 13.5% in those non-eligible. The remaining respondents (7; 5, 9%) were neutral. Of respondents, 90% were willing to accept PEPV; this was 90% in those PPV eligible and 90% in those non-eligible (Table 2).

**Table 2:** Proportion of respondents who reported (certainly or likely) willing to accept vaccination when offered, those who were neutral, and those who were reporting (likely or certainly) not willing to accept vaccination after being invited, by population subgroups, GBMSM/TGP participating in Dutch online mpox-survey (22 July-5 Sept 2022).

	**Entire study population** ***N*** = **1,856**			**Eligible for vaccination& (*****N*** = **938)**			**Non-eligible for vaccination& (*****N*** = **918)**		
	**Willing to accept**	**Neutral**	**Unwilling**	**Willing to accept**	**Neutral**	**Unwilling**	**Willing to accept**	**Neutral**	**Unwilling**
	**% (** ***n*** **)**	**% (** ***n*** **)**	**% (** ***n*** **)**	**% (** ***n*** **)**	**% (** ***n*** **)**	**% (** ***n*** **)**	**% (** ***n*** **)**	**% (** ***n*** **)**	**% (** ***n*** **)**
Willing to accept PPV	81.5% (1,512)	6.9% (128)	11.6% (216)	85.2% (799)	5.0% (47)	9.8% (92)	77.7% (713)	8.8% (81)	13.5% (124)
	(1,132 certainly, 380 likely)		(70 certainly, 146 likely)	(666 certainly, 133 likely)		(30 certainly, 62 likely)	(466 certainly, 247 likely)		(40 certainly, 184 likely)
Willing to accept PEPV	89.8% (1,667)	4.5% (83)	5.7% (106)	90.0% (844)	4.2% (39)	5.9% (55)	89.7% (823)	4.8% (44)	5.6% (51)
**By characteristics**, **Supplementary material 2**									
**Sociodemographics**									
**Country of birth**									
The Netherlands	81.6 (1,483)	7.5 (128)	12.6 (216)	83.8 (648)	5.7 (441)	10.5 (81)	75.9 (600)	9.4 (74)	14.7 (116)
Other	90.1 (264)	3.4 (100)	6.5 (19)	91.5 (151)	1.8 (3)	6.7 (11)	88.3 (113)	5.5 (7)	6.3 (8)
**Residence country**									
The Netherlands	81.6 (1,512)	6.9 (128)	11.5 (216)	85.4 (689)	4.9 (45)	9.7 (89)	77.7 (698)	8.9 (80)	13.4 (120)
Other	29 (74.4)	7.7 (3)	17.9 (7)	73.7 (14)	10.5 (2)	15.8 (3)	75.0 (15)	5.0 (1)	20.0 (4)
**Residence level of urbanization**									
Rural areas	68.4 (80)	12.8 (15)	18.8 (22)	75.0 (42)	8.9 (5)	16.1 (9)	62.3 (38)	16.4 (10)	21.3 (13)
Hardly urbanized areas	70.8 (97)	9.5 (13)	19.7 (27)	72.3 (47)	7.7 (5)	20.0 (13)	69.4 (50)	11.1 (8)	19.4 (14)
Moderately urbanized areas	79.5 (140)	11.4 (20)	9.1 (16)	83.5 (66)	10.1 (8)	6.3 (5)	76.3 (74)	12.4 (12)	11.3 (11)
Strongly urbanized areas	79.9 (306)	6.0 (23)	14.1 (54)	83.3 (155)	5.9 (11)	10.8 (20)	76.6 (151)	6.1 (12)	17.3 (34)
Extremely urbanized areas	86.6 (844)	4.9 (48)	8.5 (83)	89.9 (463)	2.7 (14)	7.4 (38)	82.8 (381)	7.4 (34)	9.8 (45)
Unknown/abroad	66.2 (45)	13.2 (9)	20.6 (14)	70.3 (26)	10.8 (4)	18.9 (7)	61.3 (19)	16.1 (5)	22.6 (7)
**Residence SES score neighborhood**									
Low	79.2 (481)	7.6 (46)	13.2 (80)	84.1 (259)	5.2 (16)	10.7 (33)	74.2 (222)	10.0 (28)	10.7 (6)
Middle	80.0 (475)	7.6 (45)	12.5 (74)	83.9 (203)	6.1 (17)	10.0 (28)	76.4 (240)	8.9 (28)	14.6 (46)
High	87.3 (495)	4.6 (26)	8.1 (46)	89.7 (271)	3.0 (9)	7.3 (22)	84.5 (224)	6.4 (17)	9.1 (24)
Unknown	69.3 (61)	12.5 (11)	18.2 (16)	70.8 (34)	10.4 (5)	18.8 (9)	67.5 (27)	15.0 (6)	17.5 (7)
**Personal received education**									
Low	75.8 (91)	13.3 (16)	10.8 (13)	81.3 (52)	7.8 (5)	10.9 (7)	69.6 (39)	19.6 (11)	10.7 (6)
Medium	77.1 (270)	8.6 (30)	14.3 (50)	76.8 (136)	7.9 (14)	15.3 (27)	77.5 (134)	9.2 (16)	13.3 (23)
High	83.0 (1,151)	5.9 (82)	11.0 (153)	87.7 (611)	4.0 (28)	8.3 (58)	78.4 (540)	7.8 (54)	13.8 (95)
**Age (in years)**									
16–30	78.9 (322)	8,8 (36)	12.3 (50)	81.9 (131)	5.0 (8)	13.1 (21)	77.0 (191)	11.3 (28)	11.7 (29)
30–45	80.0 (485)	6.3 (38)	13.7 (83)	84.1 (275)	5.2 (17)	10.7 (35)	75.3 (210)	7.5 (21)	17.2 (48)
45–55	83.2 (313)	5.1 (19)	11.7 (44)	86.6 (187)	4.2 (9)	9.3 (20)	78.8 (126)	6.3 (10)	15.0 (24)
>55	84.1 (392)	7.5 (35)	8.4 (39)	87.7 (206)	5.5 (13)	6.8 (16)	80.5 (186)	9.5 (22)	10.0 (23)
**Medical factors**									
**Used HIV-PrEP**									
No	80.7 (1,102)	7.2 (98)	12.2 (166)	86.8 (389)	3.8 (17)	9.4 (42)			
Yes	83.7 (410)	6.1 (30)	10.2 (50)	83.7 (410)	6.1 (30)	10.2 (50)			
**HIV status**									
Negative/not tested or non-disclose	80.8 (1,318)	7.4 (120)	11.8 (193)	84.9 (605)	5.5 (39)	9.7 (69)			
Positive	86.2 (194)	3.6 (8)	10.2 (23)	86.2 (194)	3.6 (8)	10.2 (23)			
**STI in past year**									
No/ don't know	80.1 (1,208)	7.4 (112)	12.5 (189)	84.1 (525)	5.3 (33)	10.6 (66)		
Yes	87.6 (304)	4.6 (16)	7.8 (27)	87.3 (274)	4.5 (14)	8.3 (26)		
**Visited public health CSH clinic in past year**									
No	79.6 (801)	8.0 (80)	12.4 (125)	85.2 (218)	4.3 (11)	10.5 (27)	77.7 (583)	9.2 (69)	13.1 (98)
Yes	83.6 (711)	5.6 (48)	10.7 (91)	85.2 (581)	5.3 (36)	9.5 (65)	77.4 (130)	7.1 (12)	15.5 (26)
**Vaccinated small pox**									
No/don't know	81.8 (830)	6.4 (65)	11.8 (120)	85.7 (433)	4.8 (24)	9.5 (48)	77.8 (397)	8.0 (41)	14.1 (72)
Yes	81.1 (682)	7.5 (63)	11.4 (96)	84.5 (366)	5.3 (23)	10.2 (44)	77.5 (316)	9.8 (40)	12.7 (52)
**Overall rated health**									
Not good/neutral	81.1 (257)	7.3 (23)	11.7 (37)	80.8 (118)	6.2 (9)	13.0 (19)	81.3 (139)	8.2 (14)	10.5 (18)
(very) good	81.5 (1,255)	6.8 (105)	11.6 (179)	86.0 (681)	4.8 (38)	9.2 (73)	76.8 (574)	9.0 (67)	14.2 (106)
**Had mpox since May 2022**									
No/don't know	81.4 (1,471)	7.0 (126)	11.7 (211)	85.4 (770)	5.0 (45)	9.6 (87)	77.4 (704)	8.9 (81)	13.6 (124)
Yes	84.4 (38)	4.4 (2)	11.1 (5)	80.6 (30)	5.6 (2)	13.9 (5)	100.0 (9)	0.0 (0)	0.0 (0)
**Social environment**									
**Know someone with mpox**									
No	79.5 (1,198)	7.8 (117)	12.7 (191)	82.6 (557)	5.6 (38)	11.7 (79)	77.0 (641)	9.5 (79)	13.5 (112)
Yes	89.7 (314)	3.1 (11)	7.1 (25)	91.4 (242)	3.4 (9)	4.9 (13)	83.7 (72)	2.3 (2)	14.0 (12)
**Know someone who had been vaccinated**									
No	76.0 (879)	9.1 (105)	15.0 (173)	79.2 (408)	6.8 (35)	14.0 (72)	73.4 (471)	10.9 (70)	15.7 (101)
Yes	90.6 (633)	3.3 (23)	6.2 (43)	92.4 (391)	2.8 (12)	4.7 (20)	87.7 (242)	4.0 (11)	8.3 (23)
**Interpersonal trust (have many people I can trust)**									
Not (completely agree)	82.1 (483)	7.8 (46)	10.0 (59)	83.9 (251)	5.7 (17)	10.4 (31)	80.3 (232)	10.0 (29)	9.7 (28)
(Completely) agree	81.2 (1,029)	6.5 (82)	12.4 (157)	85.8 (548)	4.7 (30)	9.5 (61)	76.5 (481)	8.3 (52)	15.3 (96)
**Share of MSM in friend-social network**									
None/some/neutral	76.5 (727)	9.2 (87)	14.3 (950)	80.2 (324)	5.4 (22)	14.4 (58)	73.8 (403)	11.9 (65)	14.3 (78)
(very) large part	86.6 (785)	4.5 (41)	8.8 (80)	89.0 (475)	4.7 (25)	6.4 (34)	83.3 (310)	4.3 (16)	12.4 (46)
**Connectedness to gay/queer-community**									
Not connected/neutral	74.4 (594)	9.1 (73)	16.4 (131)	78.4 (280)	7.3 (26)	14.3 (51)	71.2 (314)	10.7 (47)	18.1 (80)
Connected	86.8 (918)	5.2 (55)	8.0 (85)	89.3 (519)	3.6 (21)	7.1 (41)	83.6 (399)	7.1 (34)	9.2 (44)
**Behavior**									
**Close non-sex physical contact with others**									
No	81.1 (1,153)	6.8 (97)	12.1 (172)	84.9 (601)	4.5 (32)	10.6 (75)	77.3 (552)	9.1 (65)	13.6 (97)
Yes	82.7 (359)	7.1 (31)	10.1 (44)	86.1 (198)	6.5 (15)	7.4 (17)	78.9 (161)	7.8 (16)	13.2 (27)
**Sex with men and women**									
Only with men	81.6 (1,450)	7.0 (124)	11.4 (203)	85.2 (799)	4.9 (47)	9.9 (90)	77.8 (674)	9.1 (79)	13.0 (113)
(also with) women in past 3 months	78.5 (62)	5.1 (4)	16.5 (13)	85.2 (23)	7.4 (2)	7.4 (2)	75.0 (39)	3.8 (2)	21.2 (11)
**Number of male partners past 3 months**									
0–1	67.5 (328)	10.1 (49)	22.4 (109)	73.2 (82)	4.5 (5)	22.3 (25)	65.8 (246)	11.8 (44)	22.5 (84)
2–3	84.6 (411)	7.0 (34)	8.4 (41)	85.0 (147)	6.4 (11)	8.7 (15)	84.3 (264)	7.3 (23)	8.3 (26)
4–5	88.4 (296)	6.6 (22)	5.1 (17)	86.9 (193)	6.3 (14)	6.8 (15)	91.2 (103)	7.1 (8)	1.8 (2)
>5	86.9 (477)	4.2 (23)	8.9 (49)	87.5 (377)	3.9 (17)	8.6 (37)	84.7 (100)	5.1 (6)	10.2 (12)
**Unprotected anal intercourse**									
No	80.0 (512)	7.7 (49)	12.3 (79)	83.7 (169)	5.4 (11)	10.9 (22)	78.3 (343)	8.7 (38)	13.0 (57)
Yes, with steady partner only	74.7 (310)	8.9 (37)	16.4 (68)	80.8 (118)	6.8 (10)	12.3 (18)	71.4 (192)	10.0 (27)	18.6 (50)
Yes, (also) with casual sex partners	86.1 (690)	5.2 (42)	8.6 (69)	86.8 (512)	4.4 (26)	8.8 (52)	84.4 (178)	7.6 (16)	8.1 (17)
**Practiced group sex past 3 months**									
No/not disclose	78.3 (1,061)	8.1 (110)	13.6 (184)	81.5 (468)	6.1 (35)	12.4 (71)	75.9 (593)	9.6 (75)	14.5 (113)
Yes	90.0 (451)	3.6 (18)	6.4 (32)	90.9 (331)	3.3 (11)	5.8 (21)	87.6 (120)	4.4 (6)	8.0 (11)
**Chemsex in past 3 months**									
No/not disclose	80.8 (1,139)	6.8 (96)	12.3 (174)	84.6 (511)	4.6 (28)	10.8 (65)	78.0 (628)	8.4 (68)	13.5 (109)
Yes	83.4 (373)	7.2 (32)	9.4 (42)	86.2 (288)	5.7 (19)	8.1 (27)	75.2 (85)	11.5 (13)	13.3 (15)
**Received money/goods in exchange for sex**									
No/not disclose	81.5 (1,482)	6.8 (123)	11.7 (213)	85.5 (778)	4.6 (42)	9.9 (90)	77.5 (704)	8.9 (81)	13.5 (123)
Yes	78.9 (30)	13.2 (5)	7.9 (3)	75.0 (21)	17.9 (5)	7.1 (2)	90.0 (9)	0.0 (0)	10.0 (1)
**Sexual identity**									
GBMSM	81.6 (1,494)	6.8 (124)	11.6 (213)	85.2 (791)	5.1 (47)	9.7 (90)	77.9 (703)	8.5 (77)	13.6 (123)
TGP	72.0 (18)	16.0 (4)	12.0 (3)	80.0 (8)	0.0 (0)	20.0 (2)	66.7 (10)	26.7 (4)	6.7 (1)

& based on information in the questionnaire, which is a proxi for the Dutch mpox vaccination program criteria (at time of questionnaire 22 July-5 Sept 2022).

### 3.4. Sociodemographic, medical, social, and behavioral determinants for vaccination non-acceptance

#### 3.4.1. Univariable analyses

Proportions of respondents being willing to accept PPV, neutral, or unwilling were presented for each of the determinant-subgroups in Table 2.

The odds of being unwilling/neutral (vs. being willing) were higher for those born in the Netherlands, who live in lower urbanized areas, or in lower socioeconomic status neighborhoods, who have lower educational level (for “neutral” only), or younger age (for “unwilling” only), who are HIV negative/untested (for “neutral” only), had past STI diagnosis, did not know a person with mpox or vaccinated for mpox, not had many MSM-friends in their friend-networks, lacked connectedness to the gay/queer community, had at most one recent sex partner, always used condoms/or practiced UAI only with a steady partner, or not recently had group sex (Supplementary material 5).

These associated determinants were observed in the entire population; these were also observed in PPV-eligible respondents (except for neighborhood socioeconomic status, age, HIV and STI status, and UAI) and in non-eligible respondents [except for educational level, age, and STI (HIV not evaluated)] (Supplementary material 5).

#### 3.4.2. Multivariable analyses

**Figure 2:**
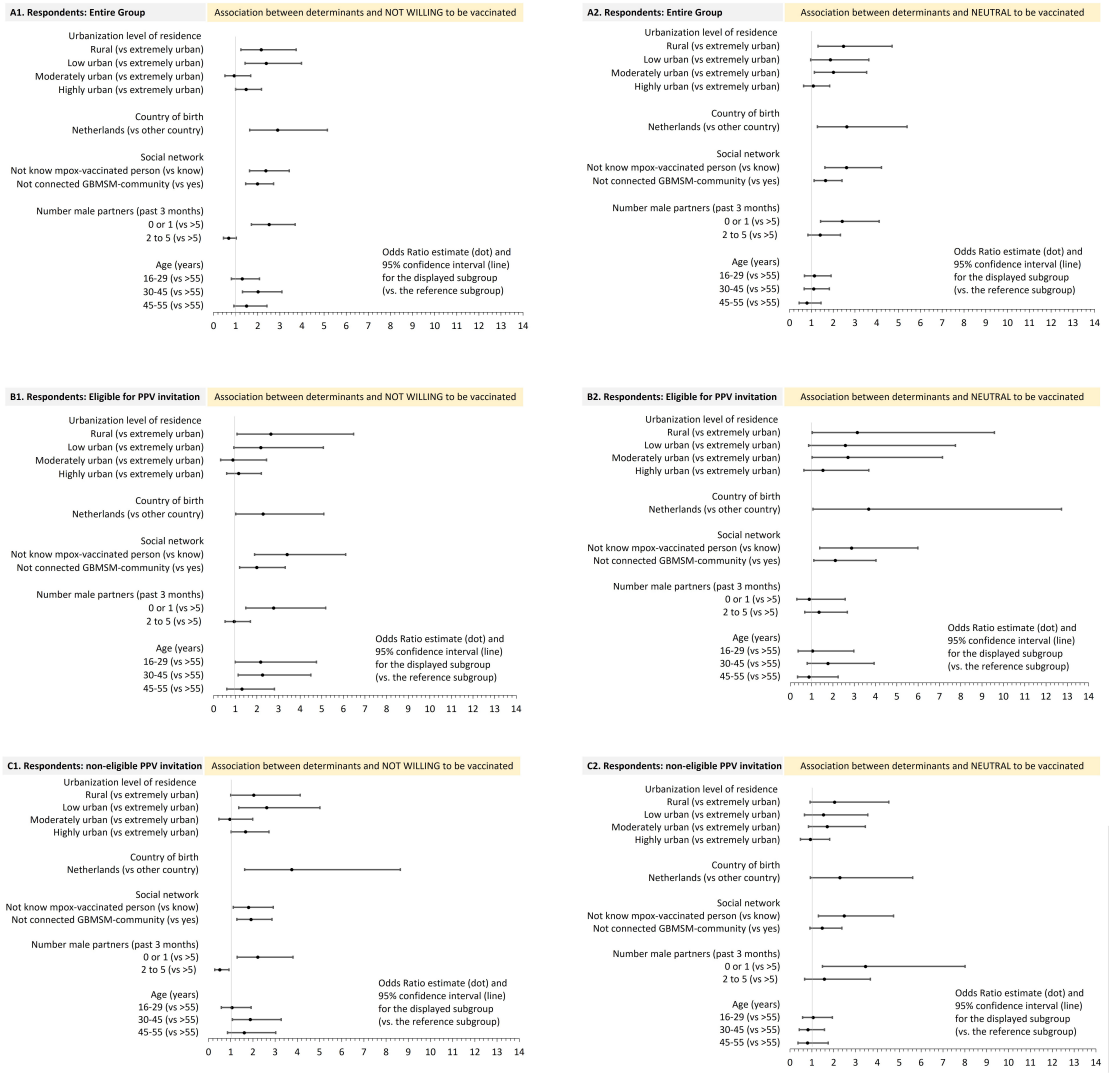
Sociodemographic, social environment, and behavioral determinants and their association with being not willing, or neutral, to accept mpox vaccination when offered, (compared to being willing), in multivariable multinominal regression analyses, mpox unvaccinated GBMSM/TGP participating in the Dutch online mpox-survey (22 July-5 Sept 2022). **(A1, A2)** entire group; **(B1, B2)** PPV eligible; **(C1, C2)** PPV non-eligible.

The odds of being unwilling/neutral to accept PPV were higher for those born in the Netherlands, who live in lower urbanized areas, not knew mpox-vaccinated people, lacked connectedness to the gay/queer community, or who had at most one recent sex partner (Figure 2). These determinants increased odds for both “unwilling” and “neutral” in the entire population and these same determinants were found associated in PPV-eligible and in non-eligible respondents (Figure 2). For those eligible, being born in the Netherlands increased odds only for “neutral” and having at most one partner increased odds only for “unwilling.” For those non-eligible, being born in the Netherlands, live in less urbanized areas [just borderline significant], or lack of connection to the gay/queer community only increased odds for “unwilling.”

### 3.5. Beliefs and their association with willingness to accept vaccination

#### 3.5.1. Beliefs distribution

Figure 2 presents the distribution of the response scores (1–5), for each belief. The proportion and number of respondents with a score of 4 or 5 were as follows:

(i) Perceived risk, severity and concern about mpox: 32.9% (*n* = 309) of eligible respondents and 13.0% (*n* = 119) of non-eligible respondents considered themselves at risk for mpox, 69.7 and 67.2% thought that symptoms could be severe, and 58.5 and 41.8% were concerned about acquiring mpox.

(ii) Motivation/importance to protect against mpox: 87.3% of PPV eligible respondents and 79.8% of those non-eligible considered it important to protect themselves from mpox, 82.8 and 71.8% considered it important to be mpox vaccinated, 93.3 and 91.1% stated that vaccinating people with high risk was a good idea.

(iii) Perceived response efficacy of mpox vaccination: 87.6% of eligible and 86.9% of non-eligible respondents expected to be protected by the vaccine, 45.1 and 45.0% (score 1 or 2) did not expect severe side effects of vaccination, 81.1 and 80.6% had trust in information about mpox vaccine as provided by Dutch institutions.

(iv) Perceived social norms: 73.0% of eligible and 67.6% of non-eligible respondents thought the gay/queer community was concerned about mpox, 72.9 and 58.3% thought that many/most in their social network considered mpox vaccination important, 70.9 and 59.0% thought many/most in their social network would get mpox vaccinated, and 30.9 and 15.5% stated that they often discussed vaccination with others.

#### 3.5.2. Association with willingness to accept vaccination

**Figure 3:**
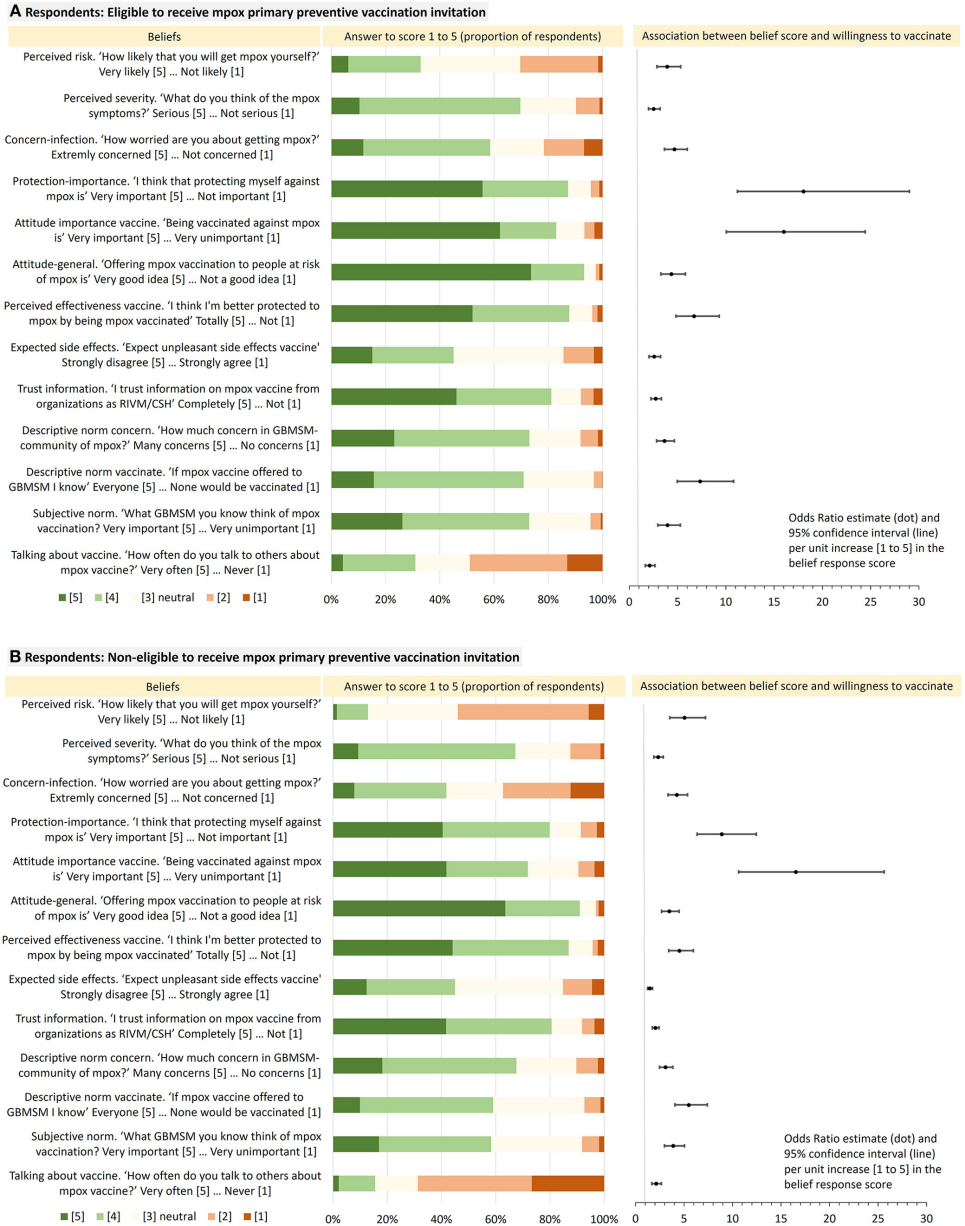
Distribution of responses to belief-statements in respondents who are eligible to receive PPV-invitation and those non-eligible **(left)**, and the odds for each of these beliefs **(right)** for their association with being willing (certainly or likely) to accept mpox vaccination when offered (compared to not willing/being neutral), in multivariable logistic regression analyses, mpox unvaccinated GBMSM/TGP participating in Dutch online mpox-survey (22 July-5 Sept 2022).

In both PPV eligible and non-eligible respondents, all evaluated beliefs were associated with being willing to accept PPV in both univariable (Supplementary material 6) and multivariable (Figure 3) logistic regression analyses. Multivariable models adjusted for country of birth, urbanization level, knowing mpox-vaccinated persons, connectedness to the gay/queer community, and number of sex partners.

### 3.6. Community advice on communicating about mpox and PPV program

A total of 1,437 (52.8%) respondents filled in the open question to give their recommendations and two main themes arose from the answers, which were communication about mpox and the PPV program and were related to (lifting) barriers regarding the access to information and to PPV.

#### 3.6.1. Communication

Respondents recommended to provide information about mpox to increase public awareness/knowledge and to improve appraisal of personal risk and severity. Respondents mentioned the importance of communicating both pros and cons of mpox vaccination, degree of protection conferred, and the development of the vaccine (“What is the vaccine made from, and history of developing it, is it safe and reliable”) and also how vaccination relates to other mpox prevention strategies (“What can we do to protect ourselves besides vaccination? Why is vaccination the best option?”). Participants suggested to also explain other mpox preventive options but not advocating abstinence. Explain the goal of the PPV-program (note: public health goal of PPV is to limit mpox spread). Such communications could stimulate a positive attitude toward mpox vaccination and increase response efficacy of mpox vaccination, as well as protection by other preventive strategies.

Respondents stated the importance of non-stigmatizing language, such as linking infection risks to behaviors (such as multiple sexual partners), not to sexual orientation (“it is not a gay disease”). It was found important to be more transparent and factual in communication (“Honest, open information, don't beat around the bush”; “Give facts, statistics”).

For optimal access to information for all people at high risk, respondents recommended to use mainstream media to underpin the importance of mpox and use public media to reach more “hidden” target groups (“Announce it publicly just like you give vaccination against COVID-19”), while others suggested community-specific channels (“Communication through gay social media”). Respondents labeled discrepant information content across channels as disruptive, and they recommended more uniformity in communication-messages across the different communication channels, also have a central website, and with more frequent provision.

#### 3.6.2. PPV-program

Respondents asked for more transparent information on who was eligible and when invited. Often respondents mentioned that the operational information around PPV was unclear and differed between healthcare providers (“It was unclear where and when I would receive my invitation for the vaccination”). They also asked to communicate who is non-eligible and what is the prospect of receiving PPV later for the non-eligible person.

Participants stated that the clarity and uniformity in both the information and in the PPV procedures across healthcare settings and geographical regions could be improved [“people who are in the PrEP program of the GGD (CSH) get priority over people who get PrEP through their GP”].

Respondents commented to improve PPV access and self-efficacy, with low-threshold options to get vaccinated. They recommended “self-registration” (“It's a pity that you can't sign up for vaccination”) in addition to personal invitations. Respondents suggested to offer vaccination at various clinic-and non-clinic settings, also outside the region of residence, including anonymous and discrete PPV options, in neighborhoods where people live (reduce travel distance), and where people get together (“Gay hangout places. But also, a mobile driving van on weekend party places. GP should be better informed and able to vaccinate and make it easier for access”), to offer vaccination at Hiv clinic, or to offer vaccination as done “with COVID-19,” “at test streets” or as is routinely done during a the CSG visit (“as with Hepatitis B”).

Respondents expressed concern that people with high risk for exposure are currently not invited for vaccination. For more inclusive vaccination-access, respondents frequently recommended to make PPV available for a broader group of people who had high risk. They mentioned to include people who not disclosed risk behavior to a healthcare professional or those who not engaged in preventive healthcare, and to offer PPV based on people's willingness, rather than on categorization of people into subgroups (“That anyone who wants a vaccine, can get the vaccine, not just HIV+ and PrEP users”). Respondents recommended to speed-up vaccination.

Other quotes regarding mpox communication and mpox PPV program are shown in Table 3.

**Table 3:** Recommendations for health-professionals and institutions to communicate on mpox vaccination and the mpox vaccination program, according to GBMSM/TGP participating in Dutch online mpox-survey (22 July-5 Sept 2022).

**Themes**	**Key illustrative comments that reflect the answers on this theme**
**Communication**	
•Non-stigmatizing communication	“Communicate risk group of people having several partners instead of only gay people” “It is not a gay disease” “It is not a sexually transmitted infection” “Stop blaming gay people with this problem. Is this the new HIV? No, it is not even an STI. You can also get it without sexual contact” “Past mistakes done in the 1980's at the time of HIV spread should not be repeated” “Media does not have to mention the target group, there are other ways to reach the target groups without explicitly mentioning them in mainstream media” “Mention impact on the broader society” “That anyone can get mpox regardless of sexual orientation or identity” “Avoid stigma of stating sexual orientation as risk group. Open vaccination to people having many partners” “Think of swingers, heterosexual couples. The emphasis is too much on man-to-man contact” “Important to mention that everybody can get mpox, not only gay people” “It is crucial to frame the message and reiterate that mpox is not an STI, and certainly not a gay disease. It's a human disease, that shall affect everyone” “There [in USA], anyone who lives in an area where it [mpox] is more prevalent or who had contact is offered a vaccination, both male and female, regardless of orientation” “Use another name for the virus that does not evoke negative associations”
•Open, transparent, clear content and language	“Honest, open information, don't beat around the bush” “Give facts, statistics” “Communicate number of how many people currently have it and how it is transmitted” “That information about the vaccine is easy to understand and in simple language” “Understandable and inclusive language” “There are so many lessons learned from COVID-19”
•Factual details such as on the vaccine, pros and cons and on mpox symptoms	“Information about the safety of the vaccine” “Information, in any case. There is too little. And information on side effects. That is what's holding me back.” “Communication about side effects” “What is the vaccine made from, and history of developing it, is it safe and reliable” “A better explanation of what the vaccination entails. Is it just a smallpox vaccine?” “Clarify that it is not actually a mpox vaccine but smallpox vaccine with 85% protection against mpox” “Degree of protection; how long protected after full vaccination” “Clearly report the risks of the disease, such a symptoms, how bad they are, how common they are” “Show pictures of skin symptoms, cigarette packaging style” “Be clear that you can be contagious 2 days prior to having symptoms, be clearer that initial flu symptoms could be monkey pox, the rash comes within days later in most cases” “Explain whether the vaccination is more important than, for example, the annual flu shot” “What happens when you get two vaccinations in a short time, of COVID-19 and mpox?” “Provide information whether previous vaccination against smallpox protects against mpox”
•Address mpox risk and severity	“How big are the risks of not taking it [the vaccine]- there are quite some contradicting opinions in press, internet and among people” “If Hiv positive undetectable, does it affect more Hiv positive persons?” “Implications of contracting the virus. the month long isolation shouldn't be discarded as ‘mild” “Stop saying symptoms are mild. The language is wrong. The symptoms are horrible, lengthy and isolating” (Risk and severity perception) “That it is transmitted by physical contact” “That you can pass it on to more people than just your sex partners” “What are the consequences if you catch the virus without vaccination and what are the consequences if you catch the virus with vaccination.” “Does it affect the rest of your life like HIV or is it temporary?”
•Explain other preventive options	“What can we do to protect ourselves besides vaccination? Why is vaccination the best option?” “Sex positive clear information” “Important to promote safe sex”
•Benefits of mpox vaccination for the community	“To reduce the risk to the community” “People who vaccinate help society in improving health of individuals and of the whole group. This is social and cost-saving” “Emphasize social responsibility” “Explain the goal of the vaccination program” “Research shows abstinence doesn't work. Therefore, the most effective intervention to slow the spread of mpox and mitigate its severity is to vaccinate the most affected communities” “Communicate that it is very important to protect yourself, but also others” “Importance of group protection” “Appeal to community-feeling” “What happens when you get two vaccinations in a short time, of COVID-19 and mpox?”
•Use various channels to disseminate information and reach people	“Communication through various media channels, radio, television, news papers, social media” “GP, Public Health Service, Television, Campaigns, National Institute for Public Health” “The government should communicate about it and openly discuss it” “Communication done by medical doctors” “Communicate broad to reach vulnerable people and avoid stigmatization” “Announce it publicly just like you give vaccination against COVID-19” “Except from MSM, it seems that little people know about the virus, the transmission routes and that it is not an STI” “STI clinic knows how to find you and how to contact you. TV and ad's on social media can reach and inform people that think mpox is an STI and only prevalent among gay men” “Communicate *via* regular media channels, but also at organizations for gay people” “Targeted information for target groups it concerns” “Communicate through gay social media” “*via* COC [community interest-organization]” “To better reach young people at high risk use social media campaign” “More visible promotional campaigns to the high risk and also ethnicity groups” “Clear information on websites and flyers. Possibility for further explanation by phone” “Explanations by phone if there are any specific questions” “Advertising *via* Instagram, for example about how and where to get the vaccine” “Use social media campaigns to reach young people” “Target groups where partners are changed more frequently. Gay saunas, sex clubs, ads on dating apps”
•Assure uniformity in information between professionals	“Make sure to inform the GP” “Involve the GPs, they are now giving wrong information” “I receive PrEP through my GP, but I didn't receive an invitation for vaccination. That is poor communication”
•Assure unity in information within and across channels	“More and clear information and education would help. A site with ALL information for example” “That all authorities tell an honest, clear and unambiguous story without stigmatizing” “There are many different stories going around” “Unambiguous. We received various messages whether we could be vaccinated...when...how. that was stressful. Do it from one organization” “The information about vaccination from the public health service *[CSH]* and RIVM is confusing”
•More information and more frequently	“Increase the amount of messages in the media that talk about mpox as I think not everyone takes this seriously” “It Is important to communicate openly and clearly. At the moment, the Government hardly paid any attention to it” “Communicate as much as possible and repeatedly” “Clarity, give regular updates, information on the status of the research” “In as many relevant languages as possible”
**Vaccination process**	
•Give clear and uniform information on triage (who is when invited)	“The predicted rollout of the vaccine should be more clearly communicated, e.g., when and where one can expect to get it” “The selection seems random. At every GGD *[CSH]* the procedure is different.” “What criteria do people have to meet to participate in the current vaccination round?” “Give information also specific for trans persons, it is unclear whether they belong to the target group of vaccination” “It was unclear where and when I would receive my invitation for the vaccination” “A time schedule who and when a vaccine can be taken” “Time between first and second shot” “I am eligible I am in the risk group, but I was not invited for vaccination. I think that more people who are in the risk group did not get a notification for vaccination”
•Give clear information on invitation process at different care providers	“I receive [HIV-]PrEP through the GP and so I'm not directly eligible for it” “U use PrEP *via* my GP and do not attend the CSH, and it is nearly impossible to get vaccinated” “I use PrEP *via* the GP and it turns out that I need to ask my GP to send my personal information [for PPV invitation] to the GGD *[SHC]* “It is ridiculous that people who are in the PrEP program of the GGD get priority of people who get PrEP through their GP” “I have Hiv and it is not clear to me whether this makes me eligible for the vaccination”
•Give perspective for those not currently invited	“Give perspective” “Communicate about the plan after the PrEP group has been vaccinated” “I would like to get a timeline for when non-high risk MSM can get the vaccine” “My friends get impatient. Some have no prospects to get vaccinated and they start to engage in risk” “I don't have access to the vaccine. Mental health is highly impacted”
•Vaccinations should be available to people with high risk of exposure, including to those not currently invited	“Important that those men who are unknown to the GGD *[CSH]* but who are in the target group, also will be given the opportunity to get vaccinated” “I test for STI at the GP and not at the CSH. Therefore, I am now non-eligible for mpox vaccine. Stupid!” “Define risk groups based on behavior, not on HIV status or PrEP use” “I have multiple sexpartners but not use PrEP and therefore cannot get vaccinated” “The selection of PrEP users is strange because the group with multiple sexpartners is much larger” “Offer to PrEP users is a good start but the population is too small”
	“Now [triage] leaves out people who practice safe sex but still have a large number of partners. This is unfortunate, since the spread of mpox is not depending on condom use” “That anyone who wants a vaccine, can get the vaccine, not just HIV+ and PrEP users” “I wonder whether other target groups should not also be invited for vaccination. Think about swingers, sex workers and bisexuals” “Quick as possible especially for men who are not ‘out’, because for them the mpox exposure is a disaster because for the outside world they are straight” “Now [triage] leaves out people who practice safe sex but still have a large number of partners. This is unfortunate, since the spread of mpox is not depending on condom use” “Allow vaccine access in the Netherlands. The situation is scary already and vaccine access is slow and harder than other EU countries” “Just as STI-care and PrEP, should mpox be offered much broader to avoid stigmatization and reduce mpox spread to zero” “The scale of it [vaccination] so small that it can hardly help” “Let people decide for themselves whether they want to be vaccinated or not”
•Lower any thresholds to get vaccinated	“Give information about practical issues” “Personal invitation is good but not reaches everyone” “Possibility to voluntary receive the vaccine” “Important to make sure that anonymous registration is possible” “It's a pity that you can't sign up for vaccination” “There should be other ways to access such as through walk-in appointment slots” “Walk in clinics at queer events, as in the UK” “My friend could vaccinate at three dates at hist CSH but that was in his holiday. He could not vaccinate at another CSH or at the GP” “A vaccine should be available free of charge to anyone who wants is, through their GP or GGD *[SCH]*” “I received an invitations for a very specific time and had to drive half an hour from my home” “I cannot choose a time and date” “Get it easily at open visiting hours” “Without appointment” “Walk-in also early evening and weekend” “I was invited, but at a very short term and at a working day. I didn't have a choice. I did not go to the vaccination appointment. I tried to reschedule but did not get a response when I called” “Free of charge” “There are no opportunities to arrange it [vaccination] on my own (I cannot for example buy a vaccine) This is very frustrating” “Paying a fee (e.g., ≤ 20€) is reasonable” “Make it [vaccination] more a national effort, because else I expect that only those who identify as gay will actually get vaccinated” “Do not fix the location, because men with multiple contacts who want to be anonymous may want to be vaccinated somewhere else, away from their residence-and living situation” “There is no means of anonymity with regard to the invitation process”
•Use various channels/locations of vaccination delivery	“At the Hiv clinic” “The easiest way would be to get vaccinated at your GP or the public health service *[SHC]*” “GP should be able to vaccinate and make it easier for access” “Would be best to get the vaccine while visiting the GGD *[CSH]* for other STD tests” “Use the same method as the hepatitis B vaccination: free of charge at parties and festivals attended by risk groups” “At cruising sites or gay saunas, many straight men have high risk sex. Thus, more at venues” “Gay hangout places. But also a mobile driving van on weekend party places. GP should be able to vaccinate and make it easier for access” “Vaccination in your own neighborhood” “Good spread of locations across the country (2/3 per province) so that is accessible also for people with various living conditions” “Leveraging COVID-19 vaccination infrastructure makes sense and is a low barrier and widely available”
•Speed up vaccination	“The most important is not to let us wait so long for the vaccine. Seems like we are the last country to offer it and it is going very slow” “As quickly as possible and easy accessible” “Explain why we are again so ‘behind’ on other countries” “Too much priority was given to the Randstad *[extremely urban areas in the Netherlands]*” “A quicker response from authorities would have been nice. Glad I was able to receive the vaccine but for a while it did feel like it was not being taken seriously as the group most affected is a minority” “Right now, it seems that the government does not see any urgency at all and does not take it that seriously”

## 4. Discussion

This survey was conducted during the 2022 multicountry mpox outbreak at the start of the national mpox PPV program in the Netherlands. This research in a convenience sample of GBMSM/TGP, assessed willingness and associated determinants for PPV (non-)acceptance, and collated respondents' recommendations on campaigning and PPV-procedures, to inform inclusive and equitable strategies in the public health response to the mpox outbreak.

### 4.1. Willingness to accept vaccination

Willingness to accept vaccination in this sample was high with 81.5% (86% in PPV eligible respondents). Another Dutch survey recruiting early July 2022 had observed 70% PPV willingness (21), which was confirmed in the current study showing 73% in the early recruitment at the end of July (Supplementary material 3). A large-scale European survey recruiting in the first half of August, observed 85–90% (northern countries) and 83–88% (western countries) (12), in line with our current Dutch study showing 86% PPV willingness in the first half of August. It should be noted that willingness fluctuated without showing an independent time trend. Fluctuations in vaccine acceptance over time are known to occur in an unfolding epidemic, with changing vaccine availability, media coverage, and number of vaccinated people and of cases over time, as was demonstrated for COVID-19 (23).

### 4.2. Optimizing communication strategies and vaccination strategies

In the evolving public health response, strategies can be further optimized to ensure broad access to information for all people, and low threshold access to vaccination for people with high risk for mpox.

#### 4.2.1. Tailoring communication messages to beliefs

The beliefs found important in mpox PPV acceptance were in line with those important in COVID-19 vaccination acceptance and in previous surveys on mpox PPV acceptance (15, 22–24). These beliefs included perceived risk/severity, motivation to protect against infection, perceived response efficacy of PPV, and perceived social norms. Of PPV-eligible respondents a third felt at risk (another third not felt at risk), over half were concerned, and majority (70%) thought that mpox symptoms could be severe. Majority of respondents (over 80%) were motivated to prevent mpox and also were positive about PPV but varied in their expectancy of side effects. Public health communication messages should include factual information on mpox (exposure risk, transmission routes, symptoms) and on the vaccine (side-effects, degree of protection for oneself and the community, history of development). This should help a person who has high risk for exposure to feel at risk and appraise mpox as potentially serious and PPV as beneficial in balance with risks of vaccinating (possible side effects) and of not vaccinating (health-and social impact of having mpox for self and others). Communications can address current uncertainties, such as around vaccine-effectiveness, and be clear that such uncertainty is well-weighted in the PPV-program choices.

People need to have access to relevant facts to be able to make an informed autonomous choice (23). Communication-messages should include information on PPV-program affairs (“who, where, and when” to increase self-efficacy to get PPV).

Communication may further address perceived social norms. Most respondents (71–73%) thought that the gay/queer community was concerned about mpox and that those people they knew deemed PPV as important and would accept PPV. It is notable that part of PPV-eligible respondents never (13%) or only sometimes (36%) discussed vaccination with others. Discussions with social network members can be helpful to encourage preventive behavior, but people might expect or experience difficulties in talking to network members about mpox prevention. Previous research on STI testing learned that people may anticipate negative reactions (public stigma) and shame (self-stigma) when discussing STI and testing, and they avoid stigma by choosing to disclose only to single/few trusted peers (selective disclosure) (25). Difficulties to discuss mpox vaccination might also arise in relation to unequal and unclear vaccine-access. It is important that information is transparent and clear about triage. Further, public health mpox communication strategies might be designed to encourage a person to talk about mpox prevention with a close trusted person and be designed to leverage possible difficulties to enable discussion of the topic in the wider community.

#### 4.2.2. Delivering messages through diverse channels

Messages can be delivered with the personal PPV invitation/reminder, which is the moment when the actual choice for PPV is made. Other channels include social media or websites which offer further benefits such as ease of maximizing dose and frequency of information exposure, and attractive (visual) tailoring to address problematic beliefs, to support decision making, norm setting, and maintenance of helpful beliefs over time (23). Most (81%) respondents in the current survey trusted information from the institutions. They recommended multiple channels to disseminate information, including mainstream media, general health websites, at clinics, at venues (where people get together), and using specific community-based channels. Respondents thereby urged to pay attention to uniformity in the information across the different channels. Information-diffusion could also be promoted by the community itself in peer-to-peer activities, although this may be less suited to reach disconnected people. Any channel chosen to reach people eligible for PPV will also reach persons currently non-eligible for PPV and thus communication should also include messages to address prevention needs of people non-eligible for PPV (explained below).

#### 4.2.3. Lifting barriers to vaccination

Low-threshold options to get vaccinated will increase the accessibility and thereby the use of PPV. To lift possible (regional-specific) barriers for PPV-invited people, respondents suggested to bring vaccination facilities close to a person's home, provide vaccination at venues where people get together, offer discrete (anonymous) vaccination, provide vaccination at the sexual health check-up, and offer the option to actively self-register for getting a vaccination, in addition to a personal invitation for PPV.

#### 4.2.4. Targeting subgroups

Subgroups less likely to want vaccination were defined by where they were born and live (born in the Netherlands, live in less urbanized areas), their social networks (no mpox-vaccinated social network members, lack of connection to a gay/queer community), and by their sexual behavior (at most one recent partner). Those who live in less urbanized areas and those who lack connection to a gay/queer community might benefit from strengthened public health efforts tailored to their context. Disparities by urbanization level in mpox PPV uptake and in other mpox mitigation strategies were demonstrated in two US studies (26, 27), and were also observed in HIV testing uptake (28). Local stakeholder networks, community based organizations and local communities can be engaged to maximize access to information (for all) and access to PPV (for those at highest risk). Respondents who lacked connectedness to the gay/queer community were less likely to want PPV, in line with research findings on HIV testing and COVID-19 testing uptake (28, 29). Disconnected people are also known to less engage in preventive healthcare. Social connectedness is a strong factor in health and driver of prevention, as people may be supported by their peers and peers can be behavioral role-models (30–32). Additional public health outreach efforts are needed to inform people who have a high risk of exposure and lack connection to a gay/queer community or to care; they may, according survey-respondents, include bisexual men, sexworkers, migrant people, very young GBMSM/TGP, and those who not disclose as GBMSM/TGP including male swingers.

### 4.3. Preventive options for people who were not invited for vaccination

People who did not receive a mpox PPV invitation had unmet needs regarding the prevention of mpox. Most PPV-non-eligible respondents in our survey did not engage in CSG/hospital care (GP was not asked for) and some reported recent group sex (15%), UAI in casual sex (23%), more than three sex partners (25%), or chemsex (12%). In the Netherlands, the PPV program aims to target people at highest risk for mpox and specifically people with multiple sexpartners. In practice, access to PPV is organized by sending personal invitation based on information as available in existing patient registries (11). While this allows to reach people in a relatively quick and feasible way, such strategy excludes those people at risk who not engaged in healthcare or had missing registry information.

Survey-respondents asked for a more inclusive PPV-access. In a US study among persons who not received mpox vaccination, more than a quarter had tried to get vaccinated (27). In current study, vaccine-non-eligible respondents showed concern about acquiring mpox (42%), wanted to protect themselves against mpox (80%), and would accept PPV when offered (78%). It is important to explore public health activities that allow low-threshold access to PPV, also for people who have high risk but who were not in existing invitation-selections.

The US and other countries had realized expansion of the number of people that can receive PPV, by application of intradermal injection, after FDA and EMA has approved its use (33, 34). However, intradermal injection will not be implemented in the Netherlands, as was stated in a recent policy brief (35). In addition to low threshold vaccine offers, well-designed communication-strategies have a crucial role in ensuring an equitable and inclusive access to information and access to preventive care options. In the challenging context of limited vaccine supply, transparent and uniform communication about triaging is especially important (36). As recommended by respondents, for all people who have a risk for exposure to mpox, regardless PPV-eligibility, information should be actively provided and easily accessible, with specific, non-stigmatizing and sex-positive guidance to enable people to act on the various mpox mitigation and care options (e.g., seek care for symptoms, and reduce close/intimate contact exposure risks) to prevent mpox acquisition, morbidity, and spread.

### 4.4. Limitations

Important subgroups, such as very young people, people with lower educational level, bisexual men, sexworkers, and TGP were underrepresented in this study, just as they are underrepresented in care. The sample is a convenience sample and not representative for all GBMSM/TGP in the Netherlands. Therefore, the main limitation is external validity, limiting generalizability of the overall proportion of willingness to vaccinate to the wider target population of GBMSM/TGP. We cannot rule out selection bias toward including respondents with a more positive attitude to PPV than the “general” GBMSM/TGP population. Strategies were taken to minimize selection bias and not to influence participation-interest or answers on beliefs, such as keeping communications in the survey text to strict a minimum. Further, selection bias might have been introduced by differential drop-out of younger people who had fewer sexual partners (Supplementary material 4). That drop-outs were more often non-accepting of PPV was in line with the more frequent PPV non-acceptance observed in respondents with few sexual partners in the survey (Table 2). Furthermore, it should be noted that overall retention in this online survey was high with 89%.

An important general limitation hampering the guidance on public health mpox preventive actions, is the lack of reliable (national, regional or subpopulation level) data on the number of people invited and vaccinated among invitees.

### 4.5. Strengths

The study sample was substantial, and respondents had representation across a variety of subgroups and geographic areas. Another strength includes the timing of the survey and the assessment of a wide range of determinants at the early start of vaccination roll-out, which provided timely data to improve preventive strategies during the mpox vaccination program. A further asset is the theoretical underpinning of the research using a combination of theoretical behavioral change models to define determinants for PPV behavior. Triangulation was applied of quantitative data on determinants and qualitative data on campaigning and procedural aspects of the PPV-program. Finally, a major strength of this study was the highly diverse and complementary composition of the research team, including scientists from fields of epidemiology, behavioral science, intervention design, implementation research, communication experts from public health centers and community-based organizations, and healthcare professionals who serve the target population. In the context of a new epidemic and its public health response, this team-collaboration made it possible to quickly collect and process data, followed by immediate communication to policy makers and those involved in mpox prevention and PPV-program activities.

### 4.6. Conclusion

**Figure 4:**
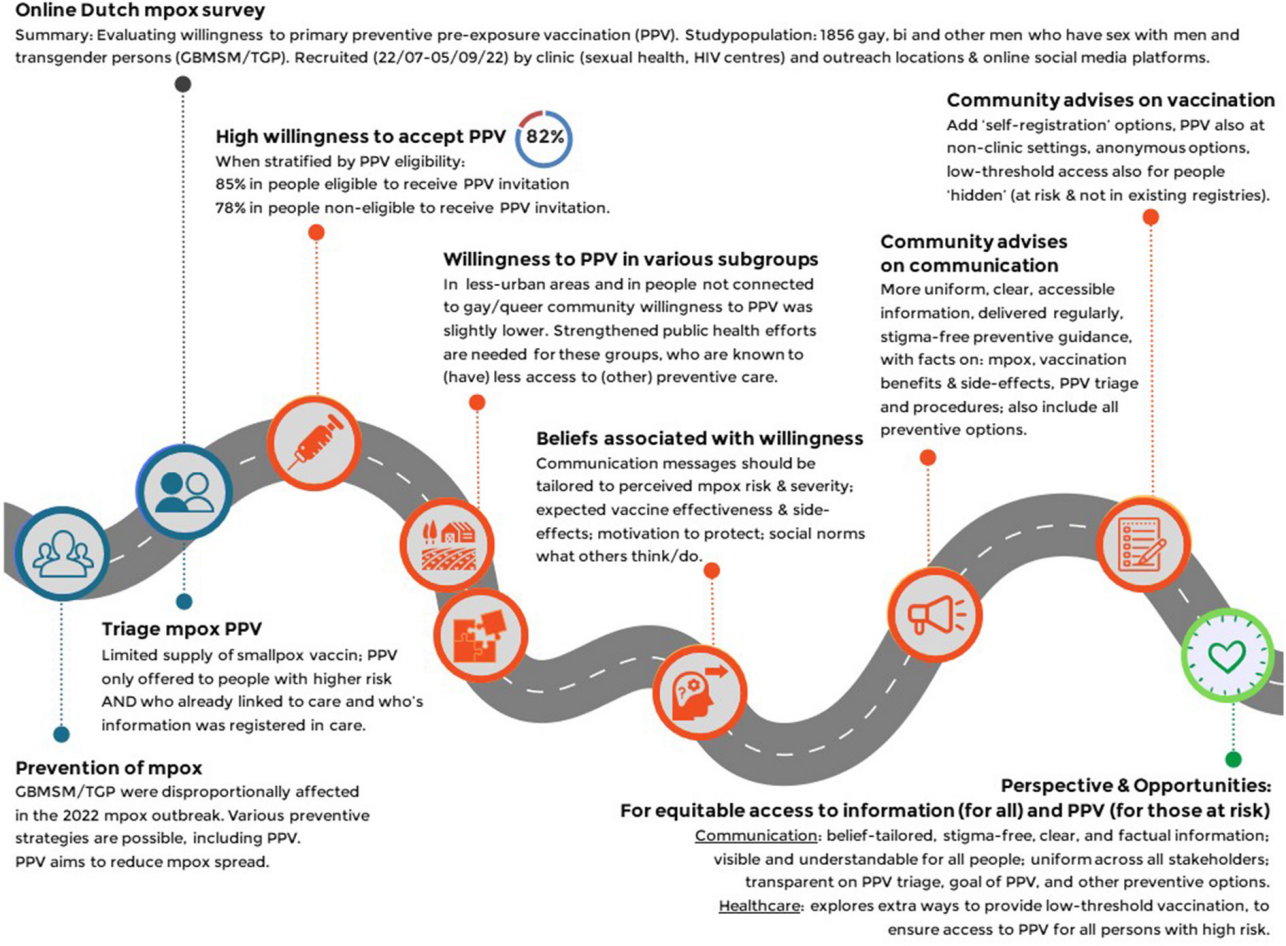
Visual summary of the study findings and conclusion. Dutch online mpox-survey (22 July-5 Sept 2022).

In the 2022 multicountry mpox epidemic, primary preventive pre-exposure vaccination of people at high risk was a key public health measure. Peoples' willingness to be vaccinated was high and they recommended low threshold options to get vaccinated, alongside clear, uniform and factual information. See for a visual summary of the findings, Figure 4. Public health efforts may be strengthened for those at risk but less likely to want vaccination, by regional approaches in less urbanized areas or outreach strategies for people who lack connection with the gay/queer community. Communication strategies will benefit from belief-tailored messages that are also transparent and uniform and provide non-stigmatizing guidance for mitigation and healthcare options. In the context of an outbreak, public health strategies should be particularly careful to maintain equitable and inclusive access to broad preventive information and care options.

## Data availability statement

The original contributions presented in the study are included in the article/Supplementary material, further inquiries can be directed to the corresponding authors.

## Ethics statement

The studies involving human participants were reviewed and approved by the Medical Ethical Committee of Maastricht University waived ethical approval because the data were coded and were analyzed anonymously (METC 2022-3324). People who were 16 years or older were eligible for participation. Participation started after providing informed consent to the study. Respondents were also asked whether they consented to be approached for later follow-up. Written informed consent from the participants' legal guardian/next of kin was not required to participate in this study in accordance with the national legislation and the institutional requirements.

## Author contributions

ND-M drafted the report and performed the statistical analyses. YE, FS, and AD'A contributed to statistical analysis. ND-M, YE, FS, CHe, A-MN, AM, UD, EH, and FS contributed to design of the survey. ND-M and YE coded the qualitative data [on respondents recommendations]. All authors reviewed the results, provided guidance, drafted, reviewed, and provided critical feedback on the report.
